# Comment on “The Brazilian Portuguese version of the psoriasis epidemiology screening tool (PEST-bp) is reliable and accurate: a cross-sectional study from southern Brazil”^☆^

**DOI:** 10.1016/j.abd.2026.501369

**Published:** 2026-05-13

**Authors:** Kishankumar Mahida, Snehal Rajendra Jagtap

**Affiliations:** a Dr. D. Y. Patil Medical College Hospital and Research Centre, Dr. D. Y. Patil Vidyapeeth (Deemed-to-be-University), Pimpri, Pune, Maharashtra, India; b Dr. D. Y. Patil Dental College and Hospital, Dr. D. Y. Patil Vidyapeeth (Deemed-to-be-University), Pimpri, Pune, Maharashtra, India

Dear Editor,

We read with great interest the study by Thomé et al., which evaluates the diagnostic performance of the Psoriasis Epidemiology Screening Tool Brazilian Portuguese version (PEST-BP) for psoriatic arthritis detection in a Southern Brazilian population.^1^ The integration of rheumatologic evaluation using the Classification Criteria for Psoriatic Arthritis (CASPAR) as the diagnostic gold standard, combined with multivariate logistic regression, constitutes a robust analytic framework. The study’s ability to validate a PEST-BP score threshold of ≥3 with high sensitivity and specificity has practical implications for dermatologic triage.

However, the reliance on a static cutoff score without stratified performance evaluation across different patient subgroups may limit interpretability in heterogeneous clinical settings.^2^ For example, patients with isolated nail disease or early-onset plaque psoriasis may not present with the systemic features that drive higher PEST-BP scores, potentially delaying referral.^3^ Incorporating differential positive predictive values for subsets such as nail psoriasis or dactylitis-dominant cases could enhance the tool’s discriminatory capacity in routine care.

Another important concern is the absence of calibration assessment between PEST-BP responses and actual joint involvement patterns. Although the authors report dactylitis and nail psoriasis as significantly more prevalent in patients with psoriatic arthritis, it is unclear whether these variables were independently associated with elevated PEST-BP scores or acted as confounders in the logistic model. Without such calibration analysis, the strength of PEST-BP as a proxy for early joint inflammation remains partially inferential.

Finally, while the authors rightly emphasize the association between higher Psoriasis Area and Severity Index (PASI) scores and psoriatic arthritis, the inclusion of PASI ≥10 as an independent predictor raises a potential conflation between skin severity and joint risk.^4^ Future investigations may benefit from dissecting whether PASI exerts an additive or synergistic effect alongside PEST-BP in composite screening models, especially in populations with low baseline disease activity.

We commend the authors for advancing localized validation of a time-efficient screening tool and for integrating dermatologic and rheumatologic expertise within the same clinical setting. Expanding this work through multicenter cohorts and longitudinal validation could meaningfully inform early intervention strategies and reduce diagnostic delays in psoriatic arthritis.

## Funding

No funding was received for this study.

## Financial support

None declared.

## Research data availability

Does not apply.

## Authors’ contributions

Kishankumar Mahida: Conceptualization, Methodology, Writing - original draft, Writing - review & editing. Snehal Rajendra Jagtap: Validation, Supervision, Project administration, Writing - original draft, Writing - review & editing.

## Conflicts of interest

None declared.
